# Age of the magma chamber and its physicochemical state under Elbrus Greater Caucasus, Russia using zircon petrochronology and modeling insights

**DOI:** 10.1038/s41598-023-36793-y

**Published:** 2023-06-15

**Authors:** I. N. Bindeman, O. E. Melnik, M. Guillong, I. S. Utkin, J.-F. Wotzlaw, A. K. Schmitt, R. A. Stern

**Affiliations:** 1 Earth Sciences, University of Oregon, Eugene, OR USA; 2 Institute of Mechanics, Lomonosov Moscow State University, Moscow, Russia; 3 Institut des Sciences de la Terre (ISTerre), University Grenoble Alpes, Grenoble, France; 4 Department of Earth Sciences, ETH Zurich, Zurich, Switzerland; 5 Department of Civil, Environmental and Geomatic Engineering, ETH Zürich, Zürich, Switzerland; 6 Institut für Geowissenschaften, Universität Heidelberg, Heidelberg, Germany; 7 John de Laeter Centre, Curtin University, Bentley, WA Australia; 8 Canadian Centre for Isotopic Microanalysis, University of Alberta, Edmonton, Canada

**Keywords:** Natural hazards, Volcanology, Geochemistry

## Abstract

Mount Elbrus, Europe's tallest and largely glaciated volcano, is made of silicic lavas and is known for Holocene eruptions, but the size and state of its magma chamber remain poorly constrained. We report high spatial resolution U–Th–Pb zircon ages, co-registered with oxygen and hafnium isotopic values, span ~ 0.6 Ma in each lava, documenting magmatic initiation that forms the current edifice. The best-fit thermochemical modeling constrains magmatic fluxes at 1.2 km^3^/1000 year by hot (900 °C), initially zircon-undersaturated dacite into a vertically extensive magma body since ~ 0.6 Ma, whereas a volcanic episode with eruptible magma only extends over the past 0.2 Ma, matching the age of oldest lavas. Simulations explain the total magma volume of ~ 180 km^3^, temporally oscillating δ^18^O and εHf values, and a wide range of zircon age distributions in each sample. These data provide insights into the current state (~ 200 km^3^ of melt in a vertically extensive system) and the potential for future activity of Elbrus calling for much-needed seismic imaging. Similar zircon records worldwide require continuous intrusive activity by magmatic accretion of silicic magmas generated at depths, and that zircon ages do not reflect eruption ages but predate them by ~ 10^3^ to 10^5^ years reflecting protracted dissolution–crystallization histories.

## Introduction

The estimation of volcanic hazards is based on a variety of tools and almost always includes imaging of upper crustal magma reservoirs that feed eruptions, as well as estimating the conditions in the magma chamber (e.g.^1^ references therein). To understand the state of the magma chamber under magmatic centers, geophysical methods are often employed^2,3^ but they alone often fail to detect liquid-dominated magma bodies in crustal subvolcanic settings, unless these exceed in thicknesses of the order of ~ 10^2^  to 10^3^ m which is on the order of the wavelength of seismic waves used in investigations. This was the case in 2009 when the 2.1 km deep Iceland Deep Drillhole Project drillhole entered hot and nearly crystal-free rhyolite 2 km below the surface in previously well-monitored Krafla caldera; the rhyolitic sill was only detected post-factum in 2015 by a specialized geophysical reflection study^4^. What kind of magma plumbing pathways and magma bodies exist under tall, magmatically productive, and commonly glaciated stratovolcanoes is still a matter of significant uncertainty.

Potentially, a combination of geophysical methods with volcanological and geochemical investigations of specific volcanoes targeting zircon petrochronology, melt inclusions, and zoning patterns in the crystalline cargo of recent volcanic products can reveal temperatures, depths, and physical state of magma bodies^1,5^. Recent efforts using zircon petrochronology (multi-methods of zircon dating and investigating ages and isotopic and chemical values) in magmatic records can shed much light on the timing of magmatic events and their pre-eruptive compositional evolution^6–15^. Diverse scenarios emerged over the last decade: in some cases, zircon age distributions and compositions are uniform and record a brief episode of crystallization in an evolved and shallow reservoir prior to eruption, but in many long-lived stratovolcanoes and calderas in continental island arcs, a more protracted record of zircon U-Th and U-Th-Pb ages provide insight into the prehistory of its crystallization, mixing and melt segregation from the crystalline residue during magmatic accretion^8,13,14,16^. Further combination of zircon ages with O and Hf isotopes as well as trace elemental ratios measured in co-registered spots within the same zircon crystals provide critical information and constraints on contributions from mantle and crustal sources, including hydrothermally-altered wall rocks, to each zircon within the magmatic system^17^. For example, some systems display extreme O and Hf heterogeneity despite similar age^14^ requiring pre-eruptive batch assembly of concurrently generated zircon-saturated and zircon bearing melts with diverse O and Hf sources, in other cases long-tailed U–Th or U–Pb ages with a relatively homogenous O and Hf isotopes requires sampling a single well-mixed, long-lived reservoir^12^.

Modeling efforts that involve forward thermochemical and thermomechanical modeling can support and augment zircon geochronological and geochemical data and constrain the size and physical conditions in subvolcanic magma reservoirs. When performed at high resolution and at realistic initial and boundary conditions that are characteristic of each volcano, the outputs of these models can be checked against geological and petrological constraints, and fine-tuned for specific situations (e.g.,^18^). Furthermore, for young and geophysically-imaged magma systems, modeling efforts can be tuned up further to constrain magmatic fluxes, temperature, and composition of intruded melts and ambient crust, and these can be compared with geophysical information about the depth of magma generation and sizes of inferred magmatic reservoirs^1,17,19^.

**Figure 1:**
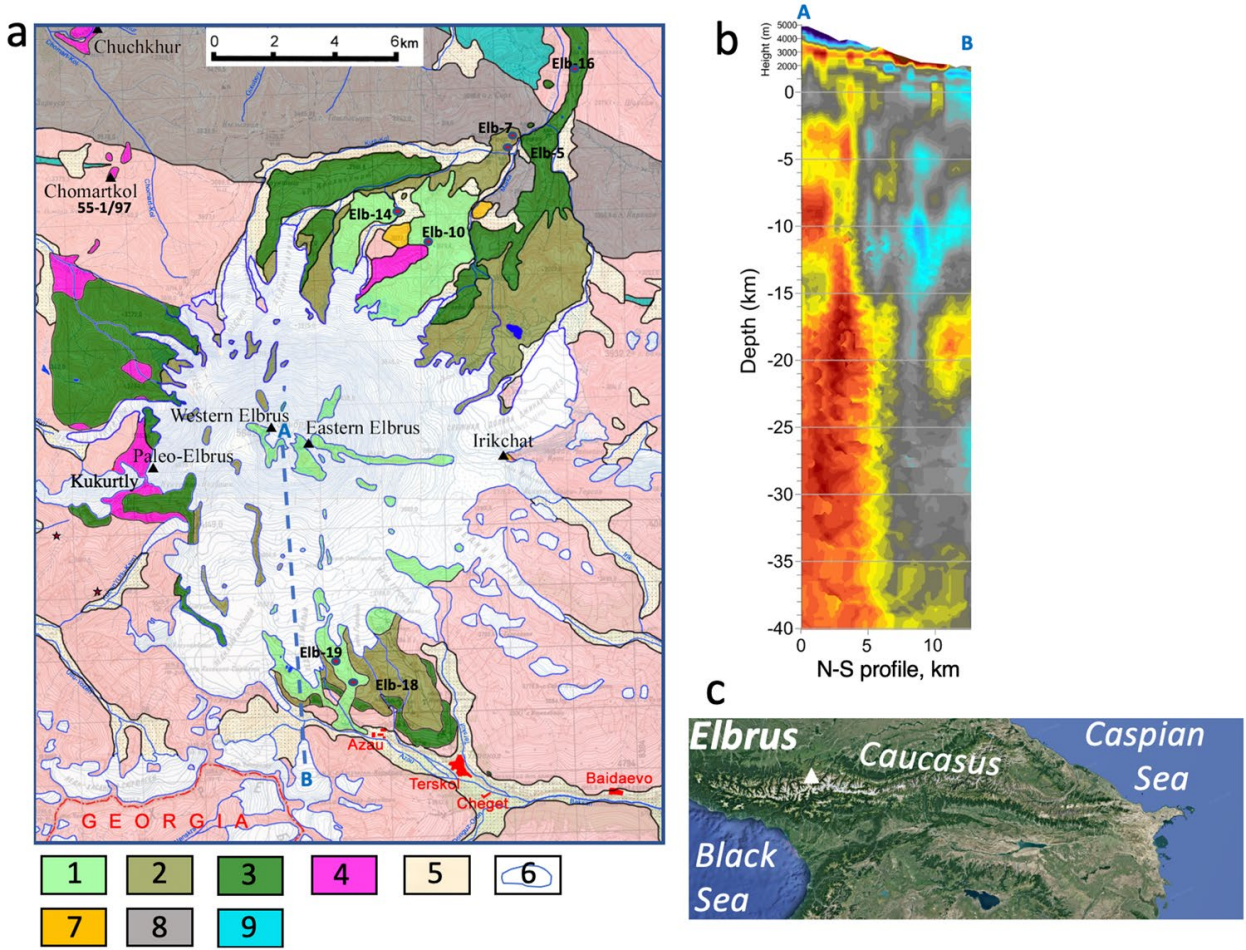
A geologic map of Elbrus volcano (Greater Caucasus, Russia) showing outlines of lavas studied here. Base map is from ref.^20^ with data from^21,22^. Elbrus Lavas (1–3); 1: < 30 ka; 2: 110–70 ka; 3: 225–170 ka; 4: Paleo Elbrus ignimbrite and lavas (900–680 ka); 5: Quaternary sediments; 6: Glaciers; 7: 1.98 Ma distal ignimbrites; 8: Mid-Late Paleozoic sedimentary/volcanic basement; 9: Jurassic volcano sedimentary cover (limestones, siliciclastics), (**a**) pink areas: Mid-Late Paleozoic crystalline basement (igneous and metamorphic). Generated and edited in Adobe Illustrator after^21^, available https://www.researchgate.net/publication/272087592_Geological_map_of_the_Elbrus_neovolcanic_center_Greater_Caucasus_scale_1100000_Edition_of_2011; (**b**) geophysical microseismic sounding data along shown cross section across Elbrus (after^23^) potentially showing vertical structures of an extended magmatic system. Red–blue colors indicate deviation from the regional Vs model (±8%). (**c**) Position of Elbrus in the Caucasus (generated via GoogleEarth software).

For volcanic hazard assessment, it is of course beneficial to combine all three methods: geophysical, geochemical-geochronological, and modeling into a system of interest. This effort can best constrain processes in magma chambers leading to volcanic eruptions, and thus aid in assessing the potential for future eruptions. Such a synthetic approach allows long- to medium-term prediction of volcanic hazards. This paper reports a combined petrological and modeling effort to investigate the origin of magmatism under the Elbrus volcano, Greater Caucasus, Russia (Fig. 1), where we found an exceptional zircon record that spans 0.6–0.7 Myr within each studied sample.

## Volcanism of the Greater Caucasus and the eruptive record of Elbrus volcano

Young (< 3 Ma) magmatism of the Greater Caucasus is related to a collision between the Arabian and Eurasian plates^24^ and has attracted attention for the past century (e.g.^25^). Recent mapping^25,20^, isotopic^21,26,27^, and geochronological efforts (e.g.^26,28,29^) including our recent U–Pb zircon investigation^30^ identified two regional magmatic spikes: older ignimbrites from Chegem (2.96 Ma), and granites, lavas, and potentially ignimbrites from the Tyrnyauz (1.98 Ma) areas. The 1.9 Ma ignimbrites underlie volcanic strata erupted from Elbrus but based on isotopic data (O and Hf in zircon) they have no connection to the Elbrus magma system and occur as extracaldera sheets. Elbrus with its Holocene age of magmatic products^31^, older voluminous regional ignimbrites, and lavas as young as 0.6–0.8 Ma^29^, with some suggestions that these were caldera-related^32^, is of particular importance as it is currently the volcanic center most likely to display potentially catastrophic magmatic activity. It is the tallest mountain in Europe and Russia combined, towering at 5642 m, some 1.5–2.5 km above its uplifted basement with a diameter of 18 km. The volume of its modern and prominent double-headed stratocone thus contains 80–100 km^3^ of lava, which erupted since ~ 0.25 Ma, based on the oldest dated Older Malka lava flow^20,27,29^. This translates into a modest eruption rate of 3–5 km^3^ per 1000 year. As it is heavily glaciated and connected by long and narrow river channels, it represents not only volcanic but glaciovolcanic hazards to its immediate environments and infrastructure^33^, should a large and sudden subglacial eruption occur. However, the age of the edifice and the overall timing of magmatic activity at Elbrus are mostly unconstrained, given that most of the volcanic record of the young Elbrus cone is covered by thick ice caps, and its oldest volcanic products may not be exposed. Competing hypotheses are that Elbrus is a relatively young stratocone, or alternatively a long-lived magmatic center (similar to Tyrnyauz and Chegem) with activity going back to at least 0.6–0.8 Ma, based on U–Pb zircon and K–Ar geochronology for rhyodacitic ignimbrites and lavas exposed at the western part of Elbrus^20,32^. Massive ~ 0.7 Ma ignimbrites are exposed in the western part of Elbrus, locally called “fluidizites”^34^. They reach thicknesses of 1 km and exhibit vertical welding structures exposed at Kukurtly, a glacially eroded wall in the western Elbrus (Fig. 1^20^). We presented an age for a sample of this ignimbrite (55-1/97) of 704 ka, Table S1^35^). Given Pleistocene glaciations, these fluidizite-textures likely indicate intrusion and quenching under an ice cap. Some researchers consider these ignimbrites as being derived from a caldera^32^, quoting a 17-km-wide circular gravity anomaly around Elbrus^36^, similar to neighboring Chegem^28^. However, despite subsequent glaciations having eroded much of this older volcanic record, a majority of geologists including us are not finding evidence for such a young caldera, given the lack of extracaldera ignimbrites, or caldera ring faults nearby. The youngest magmatic activity is poorly constrained, but existing ^14^C dates place the youngest flows in the north and south of the volcano into an age range of < 1 to 2 thousand years^36^.

**Figure 2:**
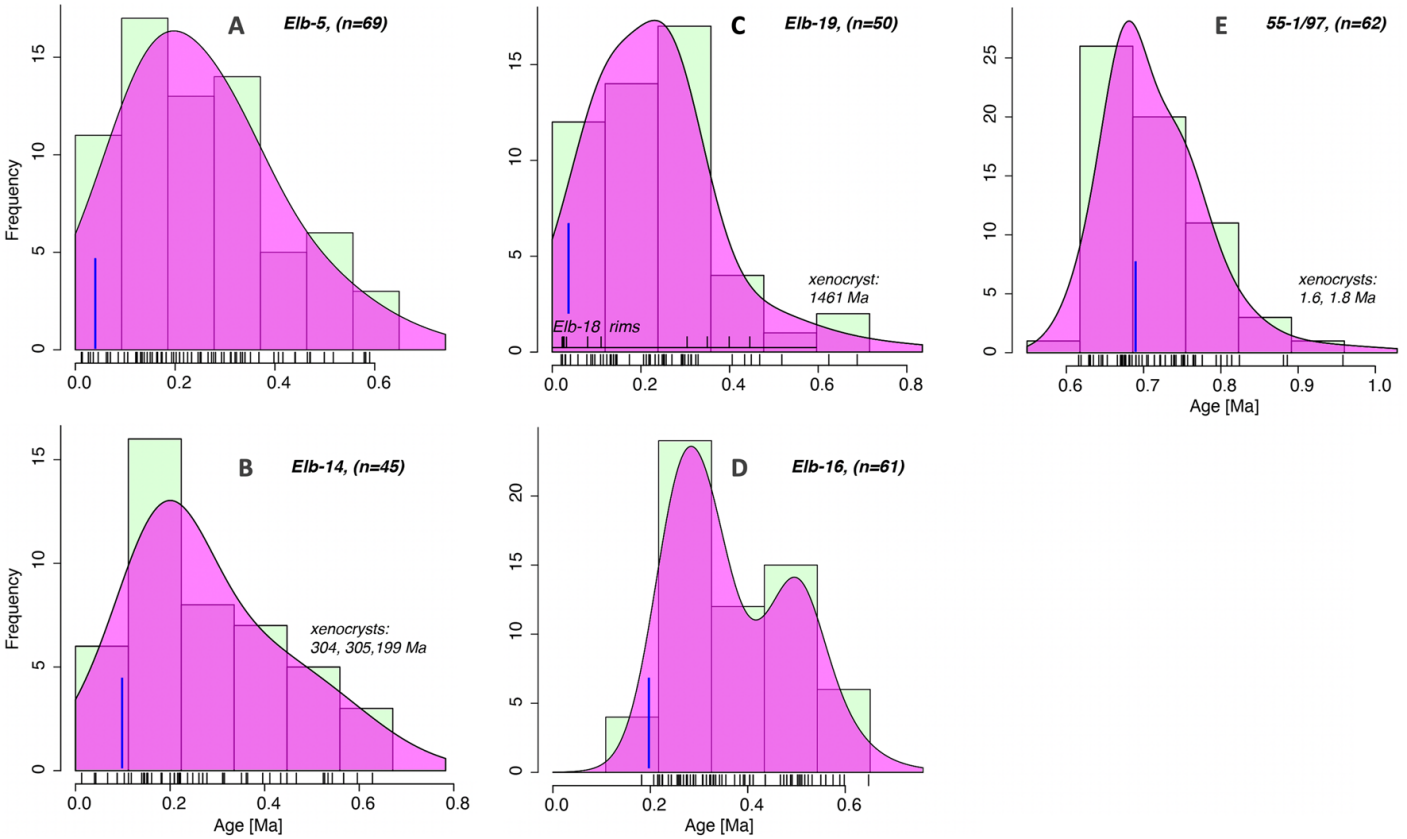
Age histograms for zircon core ages determined by LA–ICP–MS (U–Th and U–Pb combined). The inferred eruption age is indicated by a blue line. Notice that zircon cores that span the entire known interval of existence of Elbrus magma system.

We here report the first zircon-based insights into the initiation of magmatism under Elbrus, and report that studied lavas all contain a unique and continuous zircon record spanning back to ~ 0.7 Ma (Fig. 2). This study only provides information on the initiation of magmatism, but allows deeper interrogation of its origin and evolution. In this paper, we provide a combination of zircon petrochronology (ages and in situ O and Hf isotopes, Supplementary Tables S1–S4) and thermomechanical modeling to explain magmatic fluxes and evolution of the magmatic system under Elbrus to understand its current state and eruption potential.

## Results

### Analytical results

We have dated interiors of polished zircons (by U–Th and U–Pb) from four Late Pleistocene and Holocene dacitic lavas and one mid-Pleistocene ignimbrite (Figs. 2and3, Supplementary Table S3) by laser ablation inductively coupled mass spectrometry (LA–ICP–MS) and SIMS to investigate if zircon can provide insights into the magmatic evolution of Elbrus, of which most of the edifice is concealed by the glaciers.

**Figure 3:**
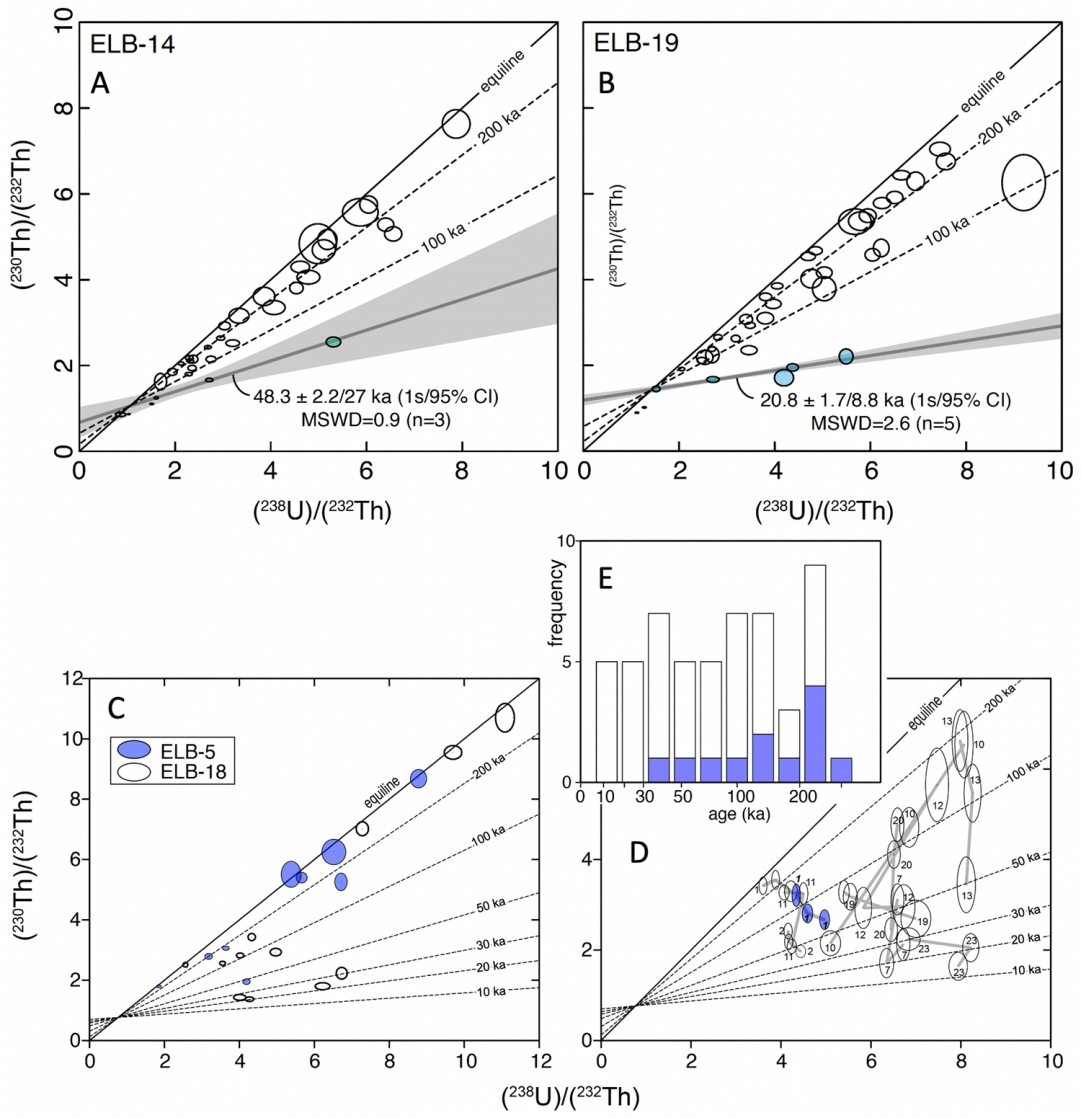
Results of U–Th dating of zircon cores ((**A,B**), by LAICPMS) and outermost rims ((**C–E**), depth profiling by SIMS). Notice significant inheritance of older zircons. In (**C**), average ages, where no significant age variability was detected with depth (~ 3 µm total depth profile); (**D**) ages calculated for ~ 1 µm outermost rim penetration in Holocene lavas, see Methods for further explanation. Numbers next to error ellipses (displayed at 1 s uncertainty) correspond to zircon number; gray lines connect progressively deeper zircon layers in each profile. (**E**) Histogram of all model ages of dated zircon rim indicating protracted zircon crystallization even for the outermost 1–3 µm of individual zircon crystals ranging between 13.9 ka and secular equilibrium (> 300 ka). Model ages are displayed for an initial (^230^Th)/(^232^Th) corresponding to whole rock Th and U abundances, assuming secular equilibrium. The younger isochron age in A and B is based on the youngest zircon cores, but these are ~ 20 to 37 kyr older than the presumed Late Holocene eruption age of these lavas, as is the zircon surface age in (**D**), suggesting that zircons were dissolving prior to the eruption (see Fig. S1).

An important result of this study is that we have discovered a continuous individual zircon age record within each studied lava of the modern Elbrus that spans from 0.7 to 0.8 Ma until 20–40 ka. Likewise, an older 0.7 Ma ignimbrite (55-1/97) at the western base of Elbrus contains zircons that span a similar age range and date back to 0.9–1.3 Ma. Additionally, among 280 dated zircons, six xenocrysts with ages 1.4–1460 Ma (Fig. 2), are found, in line with the country rocks exposed (Table S3). The xenocrysts not only have older ages, but also much different δ^18^O and εHf isotopic values, incompatible with the Elbrus’s magmas.

The youngest zircon age population dated by U–Th isochron methods ranges between 20 and 48 ka (Fig. 3, see Supplementary Table S3). However, even these youngest ages are significantly older than the inferred post-Pleistocene glacial (< 10 to 15 kyr) eruption ages of the sampled lava flows. Most of the Greater Caucasus was covered by thick ice caps during Pleistocene glaciations, morphologically reflected in U-shaped river valleys as low as 1 km elevation. For example, the surface of the Elb-14 lava flow (U–Th isochron age of 48 ka) preserves delicate pahoehoe and a–a types flow emplacement features, uneroded and unvegetated morphology, and is uncovered by moraines. Sample Elb-5 near Sultan waterfall, which was emplaced in a river valley displaying pinnacles-like structures (locally known as Arrows of Elbrus) related to water escape^37^, contains zircon with U–Th zircon ages of 20 ka as the youngest population, which are of synglacial age and thus inconsistent with postglacial river emplacement. Samples Elb-19 and 18 are the youngest lavas collected at presently glaciated slopes at 3000–3500 m elevation and have features of erosion by modern glaciers. Older Malka Lava Flow (Elb-16), dated at 0.2 Ma^27^ is more eroded, weathered, and covered by moraines. This and other young lavas yielded only zircon in secular equilibrium. We thus consider all these U–Th model ages as not reflecting the true eruption age, but rather the latest crystallization of zircon.

To further investigate the limits that zircon crystallization places on eruption ages, zircon faces in two samples (Elb-5 and Elb-18, same lava as Elb-19) were analyzed using secondary ionization mass spectrometry (SIMS) to determine the crystallization ages of the youngest rims (Fig. 3, Supplementary Table S4). Zircon faces sampled to a depth of ~ 1 µm returned > 20 kyr ages that also predate the inferred post-glacial eruption ages (Fig. 3). Although collectively zircon rim ages are younger than core ages in the same lavas, depth profiles of zircon faces reveal increasing zircon ages with depth even with minimal ~ 3 µm deep penetration (Fig. 3), and sometimes even the outermost surface ages overlap with core ages. It thus appears that zircon rims that crystallized just prior to eruption are either undetectably thin or completely absent. This may indicate that these zircons were dissolving (rather than growing) before the eruption or was shielded from the melt by storage in a solidified part of the intrusive complex, or a phenocryst as an inclusion. To interpret this result further, we extracted all zircon crystals by HF dissolution from one young lava (Elb-5) and measured the crystal size distribution of zircon using crystal lengths (Supplementary Fig. 1). There is a prominent lack of smaller crystals (< 20 μm) and a deficiency of small (< 50 µm) crystals that are consistent with the dissolution, or starved growth of this crystal population prior to eruption (e.g.^38,39^).

**Figure 4:**
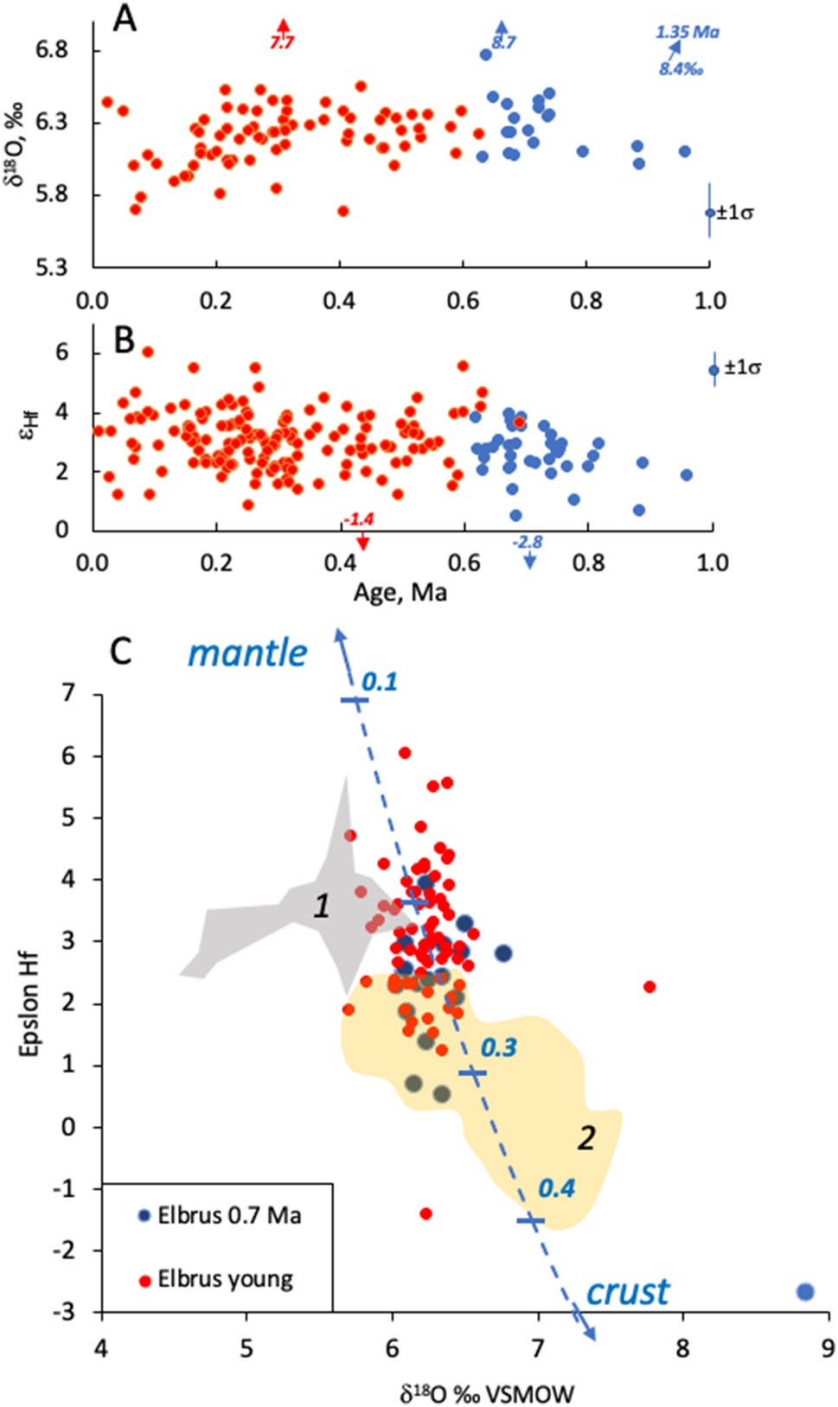
Oxygen and Hf isotopes measured in dated zircons in co-registered spots demonstrating significant within-sample heterogeneity suggesting that magmas are not tapping a single homogenous reservoir and represent about equal mix of local crustal and mantle proportions. (**A,B**) Temporal variations, (**C**) correlation of O and Hf isotopes in co-registered spots. Fields are shown for zircon from neighboring volcanic centers: 1: Chegem and Zayukovo, 2: Tyrnyauz and 1.9 Ma ignimbrites in the base of Elbrus (data from^30,35^) Mixing line showing percentage of crustal contribution is drawn between crustal (δ^18^O =  + 9‰, ε_Hf_ = − 10, 4 ppm Hf) and mantle (δ^18^O =  + 6‰, ε_Hf_ =  + 10, 2 ppm Hf ) end-members estimated as realistic for Caucasus^27,30,35^.

Oxygen and Hf isotopes were investigated in spots overlapping with U–Th or U–Pb ages (Fig. 4, Supplementary Table S2). Including all data points for Elbrus-aged zircons, O isotopes vary from + 5.6 to + 8.6‰, and ε_Hf_ from −2.8 to + 6.1, with a range of values in individual samples exceeding analytical uncertainties. An even greater isotopic range is found for zircon xenocrysts (Figs. 2, 4). Furthermore, there are also subtle temporal trends and differences with 0.7 Ma ignimbrites and other regional centers such as Chegem and Tyrnyauz.

## Discussion of analytical data

A continuous individual zircon age record (Fig. 2) going back to 0.6 Ma conveys a scenario of long-term magmatic initiation at ~ 0.6 Ma underneath the modern Elbrus cone. Lack of continuity to 0.7 Ma and older zircons found in underlying ignimbrite indicate that these are likely two unrelated episodes and independent magma systems. The presence of xenocrysts also suggests that rocks of a variety of ages were melted and incorporated preeruptively into Elbrus magmas and zircon survived dissolution. Preservation of earlier magmatic zircons from the Elbrus magmatic system, and also of older country rock-derived xenocrysts provide an insight into continuous zircon saturation, an important constraint to modeling magma reservoir processes.

Zircon in Elbrus yielded high-δ^18^O and low ε_Hf_ values, requiring a ~ 30:70% mix of local unradiogenic Hf and high-δ^18^O supracrustal materials and the mantle (Fig. 4). These values are also distinct from the neighboring Chegem ignimbrites and Tyrnyauz granites^30,34,35^. Another important feature of the zircon record is its heterogeneity with respect to Hf and O isotopes, in excess of analytical uncertainties. Compared to similar studied zircon examples worldwide, Elbrus zircons are relatively more homogenous than Yellowstone hot spot track magmas^13,14,17^. There are no low-δ^18^O zircons in Elbrus, although these are present in neighboring Chegem^30^; the presence of low-δ^18^O zircons are commonly associated with remelting of hydrothermally-altered rocks commonly found in multi-cyclic calderas. Thus, the lack of low-δ^18^O zircons in Elbrus may indirectly indicate a lack of preexisting caldera.

Given that most of these magmas are silicic (dacitic to rhyodacitic), with identical mineralogy and crystallization conditions^35^ as well as comparable Sr–Nd–Pb–Hf isotopic values^27,30^, magma production mechanisms and magma sources appear to have remained constant for over a million years. Thus, zircon petrochronology not only changes the view of the longevity of continuous magmatic activity under Elbrus but given overall compositional and zircon O and Hf isotope similarity, suggests a deep rather than shallow magma source lasting for > 1 Ma, split into two magmatic episodes.

Overall, zircon O and Hf isotopes show a subtle dilution of crustal signatures with more mantle-like compositions over 1 Ma resulting in a decrease in δ^18^O and an increase in ε_Hf_ (Fig. 4, Tables S1 and S2), whereas SiO_2_ drops ~ 4 wt% and MgO increases 1 wt% from 0.7 Ma ignimbrites to youngest Elbrus lavas. We thus prefer a scenario where silicic magmas, above or slightly below zircon saturation temperatures, intruded from depths and fed into an upper crustal magma reservoir underneath Elbrus. The crustal isotopic signatures of O and Hf are likely inherited from this deep crustal source, rather than produced by shallow crustal assimilation, because of more efficient assimilation at higher temperatures of the country-rock at greater depth, and because shallow melt production would have generated a more diverse record as observed in many other volcanoes around the world. The deep hot zone (generated near, or slightly above the Moho; e.g.^18^) likely developed as a result of contact between the juxtaposed subduction-influenced mantle and Paleozoic and older crust, in the course of lower crustal delamination in response to collision (e.g.^30^). The minor secular compositional and isotopic variations suggest that isotopically more mantle-like primitive isotopic values are carried by silicic differentiates of mantle-derived mafic magmas in hot zones. We consider these constraints in the modeling below.

## Numerical modeling of a magma chamber under Elbrus

### Set up for numerical model and hypotheses it checks

Constraints from the petrological study of Elbrus volcanic rocks include: (1) a zircon record indicating 0.6 Ma longevity of magmatism and inheritance; (2) overall (rhyo-)dacitic composition with only slight variations over time; (3) diversity of O and Hf isotopes within products of many eruptions; (4) a total estimated volume of magma produced given by the size of Elbrus edifice 18 km diameter and 2.0–2.5 km relative elevation, a minimum of 80 to 100 km^3^ of erupted dacitic lava material is present. Doubling this number to account for glacial and other erosion happening in between eruptions, as well as pyroclastic eruptions that disperse material beyond volcanic edifice, would correspond to 160–200 km^3^.

Age diversity of zircon at relative compositional and isotopic similarities of magmas in the record suggests similarity of magma genesis processes for over 0.6 Ma of magma petrogenesis under Elbrus. Oxygen and Hf isotopic values suggest 25–35% crustal contribution to mantle-derived magma (Fig. 4). Like many other central volcanoes, Elbrus exhibits a vertical magmatic system from 3 to 11 km and 15 to 45 km that is constrained by seismic and acoustic profiling (^40,23^; Fig. 1b). Magmas coming from the lower to the upper chamber must thus carry enough heat and liquid magmas to produce eruptible melts and not completely dissolve zircon from previous intrusions and xenocrysts inherited from the ambient crust. This means that the magma in the vertical magma plumbing system maintained temperatures below ~ 800 °C (the computed zircon saturation for Elbrus magmas), but rather juvenile magma was added in small but persistent increments to the resident magma, which became mixed and hybridized, producing the protracted zircon crystal age distribution observed. This suggests that the added magmas are neither strongly overheated nor compositionally very different from the resident magmas. Basaltic magma with its high temperature and zircon solubility would be more capable of aggressively dissolving preexisting zircons^9^, and additionally would have generated compositionally more diverse isotopic and chemical records, either bimodal or more continuous. A modeled example of basaltic magmas intruding into the mid-crustal area results in zircons with extremely diverse O and Hf isotopic values as is observed in Yellowstone-Snake River Plain magmas^17^. The Yellowstone plume is characterized by high basaltic magma flux rates, and an extensional crustal regime, different than the compressional regime in the Greater Caucasus.

The modeling was done using a code modelling dike and sill intrusions along with crustal melting and eruption upon reaching a melt threshold, ref.^41^). Simultaneously, the model monitors the crystallization of zircon up to the time of eruption^9^. In this modeling, we thus vary the magma flux rates (from 0.1 to 10 km^3^/1000 years to be able to generate the liquid magma output comparable to the one observed. We further varied the eruption efficiency (% of erupted magma over the total available magma with the crystal content above some critical value, from 25 to 90%), Zr concentrations within ranges observed in Greater Caucasus (^30,35^, from 170 to 220 ppm Zr), and crystal content in erupted magmas as related to temperature based on phase diagrams.

We assume an initial temperature gradient in the crust of 20 °C/km. Parameters for the simulations are specified in Table 1. Dikes are randomly intruded into the simulation domain. In the upper part of the model dikes transition to sills; as the system develops, spreading, uplift, and sagging is observed in the middle, upper, and lower parts of the system, respectively, and these are all balanced with respect to mass and heat balance. Volcanic eruptions drain certain areas in the system, and these also obey laws of mass and heat conservation.

**Table 1:** List of physical parameters used in modeling.

Parameter	Description	Value
*ρ*	Density	2650 kg/m^3^
*λ*_r_	Thermal conductivity of rock	1.5 W/m/K
*λ*_m_	Thermal conductivity of magma	1.2 W/m/K
*C*_*p*_	Specific heat capacity	1350 J/K/kg
L_*_	Latent heat of melting/crystallization	3.5 × 10^5^ J/K/kg
*T*_m_	Magma intrusion temperature	950 °C
*T*_top_	Temperature at depth *z* = 0 km	100 °C
Δ*T*	Temperature geothermal gradient	20 °C/km
*ν*	Poisson’s ratio of rock	0.3
*E*	Young’s modulus of rock	15.6 GPa
*Q*_in_	Intrusion rate	1.2 km^3^/1000 year
[*z*_min_, *z*_max_]	Dike center depth	3–24 km
*W*	Width of dike intrusion region (focusing)	5(3) km
[*zs*_min_, *zs*_max_]	Depth range of sills formation	3–4 km
*θ*	Dike rotation angles	85°–95°
*a*	Dike half length	100–1500 m
*b*	Dike half thickness	10–20 m
*h*	Dike transverse width	5 km
*δ*_*cr*_	Critical melt fraction	90%

### Results of numerical experiments

**Figure 5:**
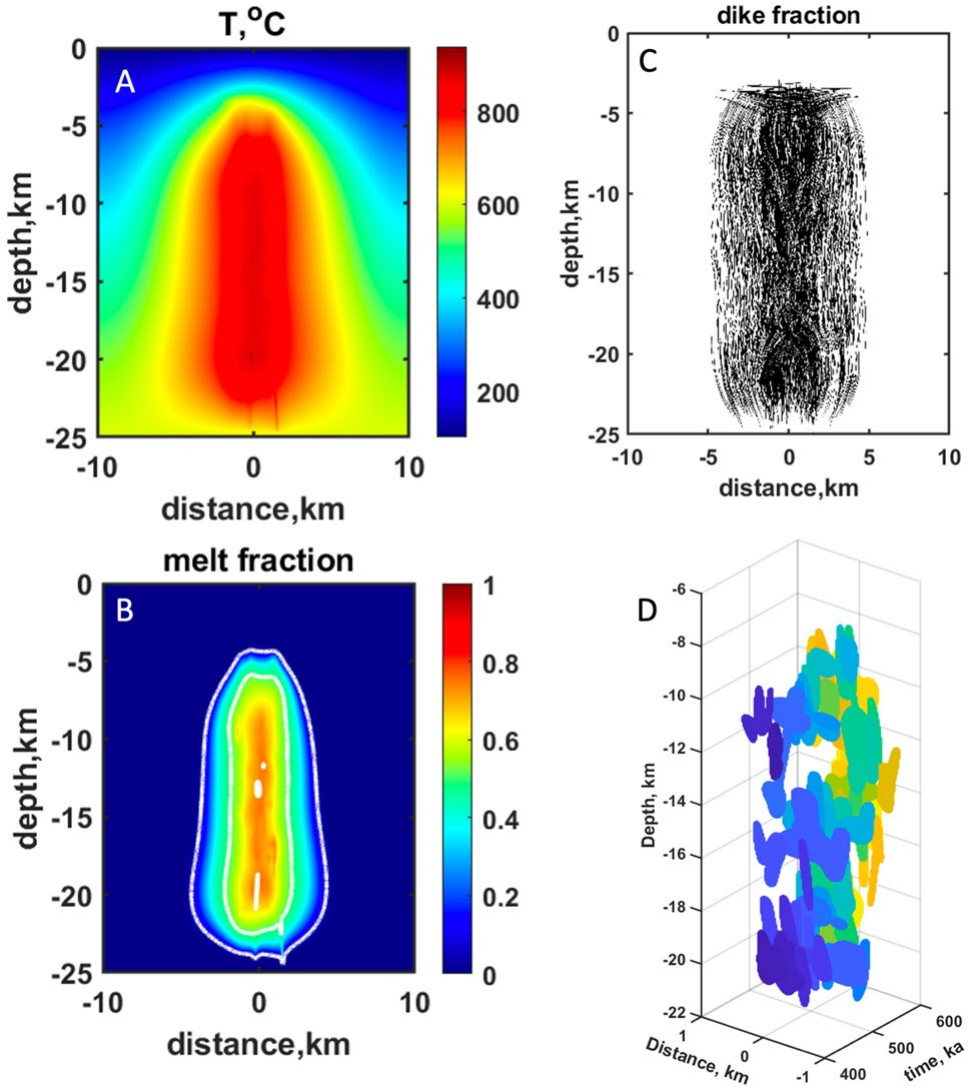
Results of Elbrus magma chamber modeling by injection of hot silicic dikes and sills for 600 kyr. (**a**) distribution of temperature; (**b**) melt fractions, contours of 5, 50 and 75 vol% melt fraction; (**c**) locations of the injected dikes and sills; (**d**) accumulated melt and the erupted volumes at different times (ka), colors connect continuous melt regions showing their complexity. It can be seen that eruptions become generally shallower with time as the system matures.

Figure 5 shows the results of best-fit simulations for the above constraints with a volumetric flux of rhyolitic magma of 1.2 km^3^ of magma per 1000 years for a total of 0.6 Ma of silicic magma injection. We assume that the initial width of the magma injection zone is 5 km, while after 300 ka, the injection area narrows to 3 km (melt focusing) as the hot zone is formed in the central part of the domain. At the end of the run, the total area affected by dike and sill injection and the resulting spreading zone corresponds to 10 km (Fig. 5c). Thus, the final dike distribution in the host rocks is twice as wide as the initial injection area due to the horizontal spreading of the system.

**Figure 6:**
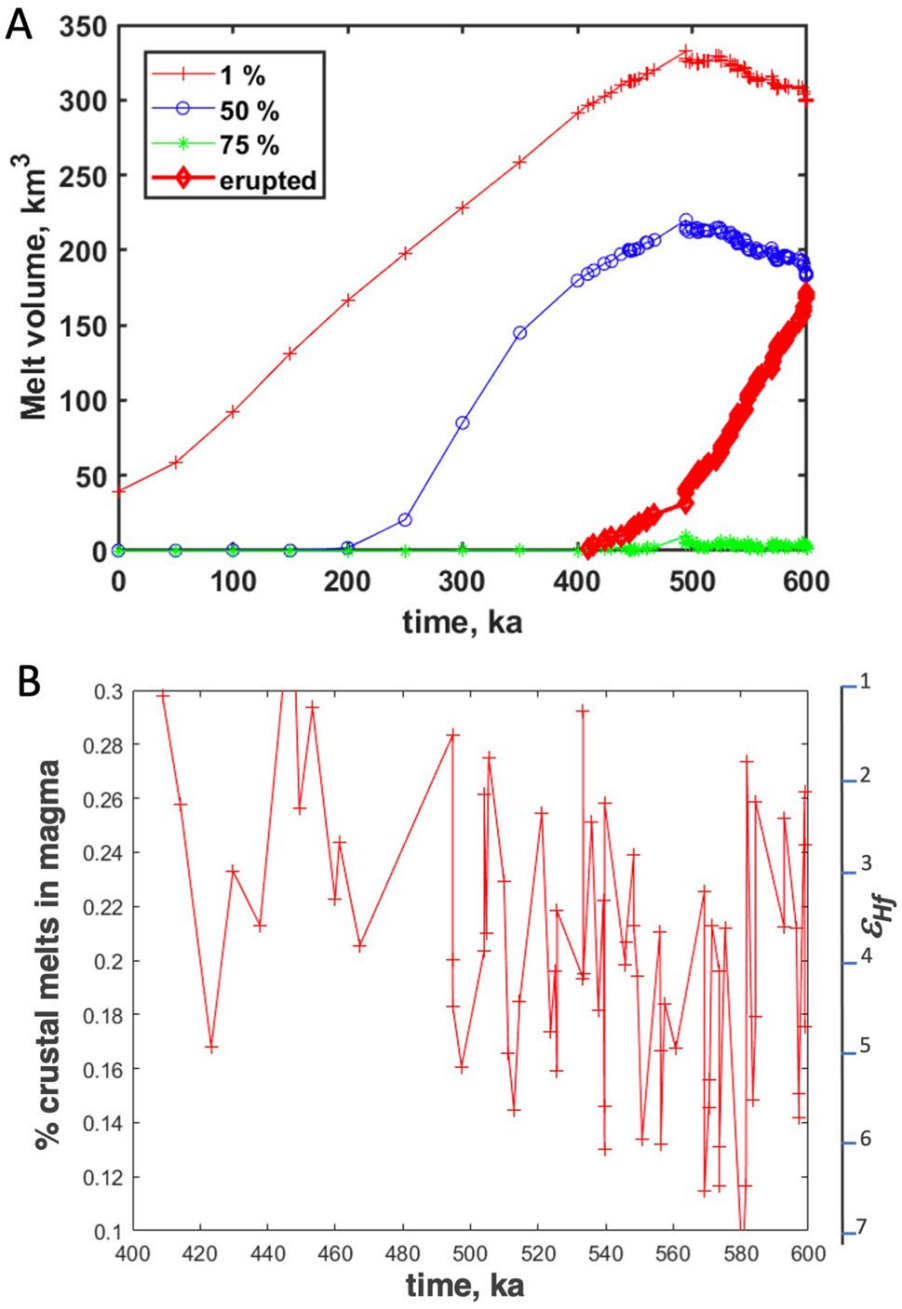
Evolution of melt volume through time. (**A**) Cumulative melt volumes formed and erupted volume. Notice an incubation time of ~ 400 kyr to form interconnected and eruptible magma bodies (Fig. 5d) with each eruption evacuating 90% of the eruptible melt. (**B**) Proportion of crustal rocks (locally melted at shallow depths) in eruptions; over the duration of magma chamber formation the fraction of crustal contribution varies between 0.1 and 0.3 (ε_Hf_ 1 to 7), and generally decreases with age, in accordance with observations (Fig. 4).

The modeling shows that after an incubation period lasting 0.4 Myrs, a hot zone with temperatures > 800 °C (Fig. 5a) is formed in the central area beneath the volcano. These temperatures would correspond to melt fractions > 80% if no eruptions are allowed in the system. However, our model considers that if a critical volume of magma with the melt fraction > 75% is formed anywhere in a vertically extensive system, an eruption occurs and removes 90% of the available magma, tapping all areas. The subvolcanic system shrinks as this occurs and mass and heat conservations are obeyed in the system^41^. We consider that eruption volumes are distributed by an exponential law^42^ as is typical for many volcanoes worldwide (more frequent small eruptions and less frequent large eruptions) and such a sequence of eruption volumes is generated randomly prior to the simulation. Larger eruptions require longer incubation intervals of melt accumulation. Figure 5b shows the distribution of the melt fraction inside the crustal domain affected by intrusion. Contours of 5, 50, and 75% of melt are shown. Model simulations show that eruptions drain most of the magma from the magma chamber while a vertically extended crystal mush zone is formed around the central part of the volcano. Melt volumes and the volume of erupted material are shown in Fig. 5d. Figure 6 presents the history of melt production and eruption and assimilation proportion of the crust in erupted material. Before eruptions start to incubate, the volume of the present melt beneath Elbrus increases progressively to ~ 300 km^3^ over 0.4 Myrs. After an incubation period, eruptions start, triggering a trend towards decreasing melt volumes as the magma is evacuated from the system to the surface forming the magmatic edifice of Elbrus. After that, continuing magma supply from depth is almost completely balanced by eruptions. The proportion of the locally melted crustal rocks in the erupted magma (Fig. 6b) ranges from ~ 0.1 to 0.3, and only slightly decreases during the evolution of the system because eruptions mingle magma from different parts of the system. This may correspond to the subtle trend of decreasing crustal contribution with time as observed for O and Hf isotopes in zircon (Fig. 4). Figure 5d shows the distribution of magma chambers with time. Their horizontal extent is much smaller than the vertical extent due to a wide range of depths of dikes injection. Eruptions start deep in the system where the thermal conditions required for melt generation are reached early, and then progressively magma drainage moves upwards as the system matures. Notice that magma bodies have complex shapes and overall would fit the current paradigm of vertically extensive magma systems^43^. Due to different melt connectivity, some eruptions sample only a narrow range of depths, whereas others excavate magma from the whole extent of the magmatic system.

We also performed model runs with periodic (oscillating) magma fluxes, but observed that eruptions stop almost immediately (with a delay of a few ka) after a pause in intrusions, while the hot interior of the magma body continues to remain hot and crystallizes slowly. Thus, there is not much difference between temporally constant vs alternating magma flux on any of the parameters discussed above.

**Figure 7:**
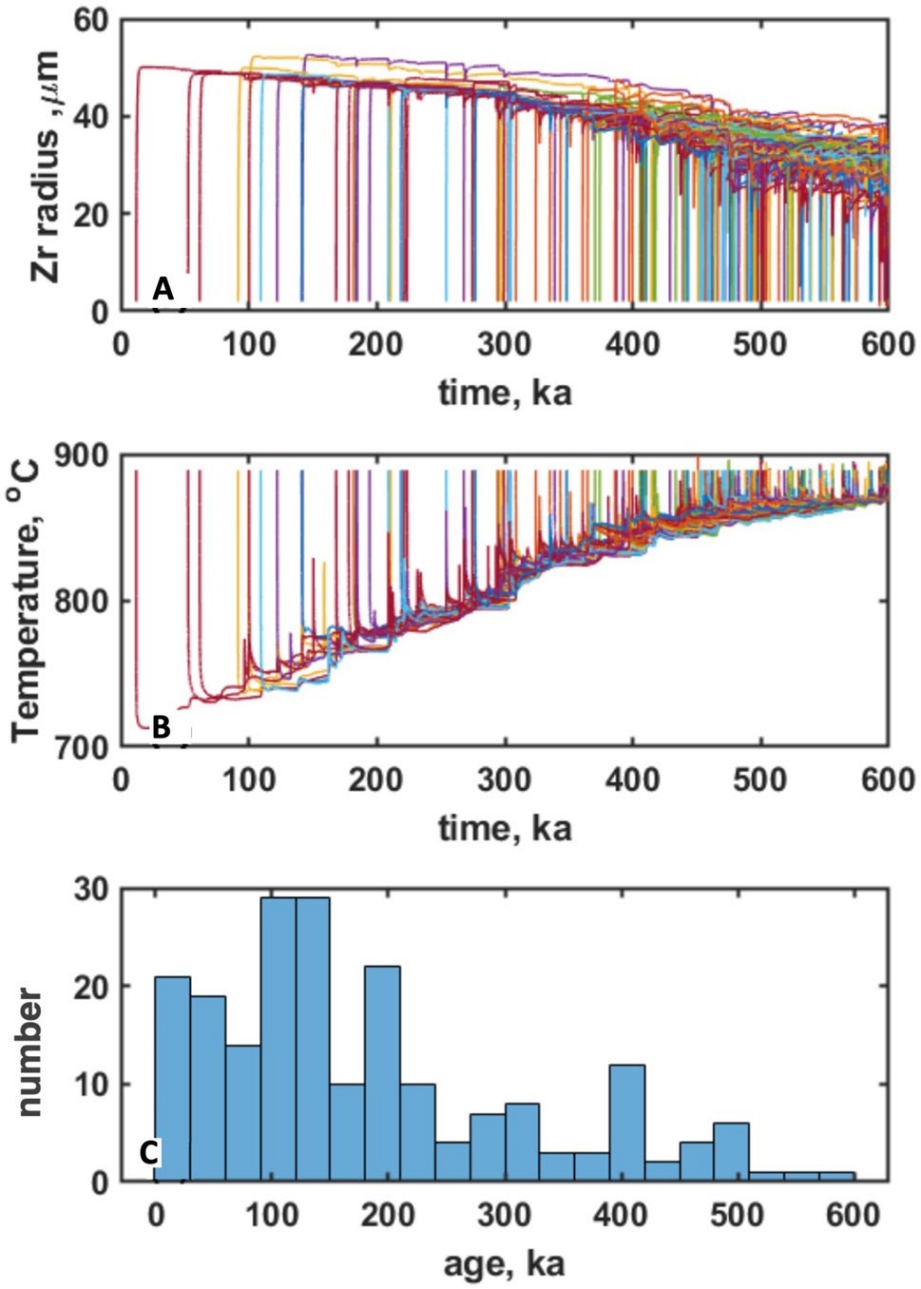
Numerically generated zircon sizes and age. (**A**) Zircon growth-dissolution episodes in zircon for different parts of the growing magmatic system as sampled by the eruptions. (**B**) Temperatures histories for these zircons. Notice that most zircon crystals first grow during cooling of dikes and then become dissolved, in line with age dating indicating that the minority of zircons is of eruption age (Fig. 3), and with zircon crystal size distributions (Fig. S1) indicating their pre-eruptive dissolution. (**C**) Histogram of zircon age distributions in products of one of the latest eruptions, where zircon crystallization ages span the entire magma formation interval, compare to Fig. 2, See Fig. S2 for more examples of numerically generated zircon inheritance.

The presented set of simulations produces the required erupted volume of the edifice at Elbrus (corrected for erosion and pyroclastic dispersal), but importantly produces a zircon record similar to the one observed (Fig. 2, 7, Fig. S2). We extract thermal histories of individual magma batches from different eruptions, and using the Bindeman and Melnik^9^ software calculated growth vs. dissolution of magmatic zircon as the system matures within each point in time and space. Typical temperature histories, zircon radius evolution, and the zircon age histogram are shown in Fig. 7 and Supplementary Fig. 2. As soon as a dike is injected into cooler host rocks, magmatic temperature drops quickly leading to a rapid increase in zircon supersaturation and consequent crystal growth. At later stages, if the volume is reheated, zircon can partially or completely dissolve and regrow. In other markers, zircon will continue to crystallize. Such conditions can be just meters apart from the dike, but the center of the magma body has reset zircon ages more. Generally, zircon dissolves during thermal maturation of the system and thus even the surface age of zircon crystals represents the oldest age corresponding to an individual dike injection time, matching our observation of the zircon surface dating (Fig. 3). Upon melt segregation, zircon from eruptible magma bodies appears in the same eruption. Thus, the modeling at different Zr concentrations supports extensive zircon inheritance in the magma system (Fig. 7c) matching the ones observed in natural samples (Fig. 2).

## Discussion

The observations and models presented here suggest that in order to reproduce 200 ka of silicic magmatism in Elbrus, corresponding to the currently known ages of its lavas, a continuing magmatic flux of ~ 1.2 km^3^/1000 years over ~ 600 kyrs is required. The record of magmatic accretion of this duration is visible in the zircon record from all eruptions in Elbrus, and is reproduced in our best-fit thermoelastic-thermochemical model that matches diverse parameters such as magma compositions, isotopic ratios, eruptive volumes, and zircon age record. We were able to generate the observed 100–150 km^3^ of lavas erupted over 200 ka by accumulating a total of 350 km^3^ of magma in a vertically narrow tube-like magma system reaching 10 km in diameter at the end of the model run. Likewise, zircon dated from 700 ka ignimbrites found in the western part of Elbrus also carry a similar record of inheritance for several 1000’s years, hinting that similar magmatic conditions characterized this earlier stage of magma system development that resulted in rather thick (1 km at Kukurtly) and voluminous ignimbrite and lava eruptions^20,30–34^. As the lavas from the modern Elbrus edifice lack any zircon dating back to the eruption ages of these older ignimbrites, we conclude that these are not a part of the same continuing magma system, temporally and/or spatially (see Fig. 1). Given that the style of magmatic accretion under Elbrus, as independently recorded by the lavas and the ignimbrites, is characterized by protracted zircon crystallization and recycling into progressively younger volcanic episodes, we suggest that current volcanism at Elbrus, forming its massive stratocone, started 600 ka. The ignimbrites record this earlier episode.

Given that a narrow and vertically extensive magma chamber is our preferred solution for Elbrus, it is perhaps geometrically permissible to separate the old (> 0.7 Ma) and new systems. This is supported by the older ignimbrites being only known from the west of the current edifice, and thus the previous magma body is likely located underneath.

Considering the future volcanic activity at Elbrus, we can learn from its prior volcanic cycle that finished at 700 ka. Model calculations show that in order to keep the Elbrus magma system active and match the zircon records, magma flux rates of ~ 1.2 km^3^/1000 years, resulting in significant (many 10 s of km^3^), and a potentially detectable amount of melt are required, suggesting a rather robust magma system under Elbrus. This paper calls for a badly needed detailed seismic investigation of the Elbrus magma chamber to better constrain its volcanic hazard potential. We suggest that very young silicic lavas of the Elbrus edifice require the presence of an active magma system with abundant liquid magma, as we have modeled. We also attest that the zircon ages do not reflect eruption ages, but predate them by ~ 10^3^–10^5^ years. This is because zircon in Elbrus, and similar young magmatic systems worldwide dated by precise U–Th methods^44–46^ and sampled by a single eruption, are both crystallizing and dissolving within short time intervals, as is evidenced by our measurements and modeling. Zircon crystals do not crystallize syneruptively, as rapid cooling and water loss during magma ascent abruptly decreases Zr diffusion rates in the melt.

Furthermore, application of our results to similar largely silicic stratocones worldwide even with moderate magma influx rates requires the presence of laterally and vertically distributed melt pockets of comparable volume to erupted material, where a higher melt fraction of 50% are concentrated in the center and record smaller tails of inheritance as compared to zircons near periphery of the system. Although our thermo-elasto-chemical model does not have an extraction, even if it did, it would likely modulate eruption-volume-time frequency (currently it is prescribed rather than self-organized) but produce only minor influence on the total melt volume. Temporal termination of magma flux from below for a few kyr significantly decreases volcanic output almost immediately, and such gap will likely result in recognizable surface erosion of the edifice, however comparable melt pockets remain in the already formed slowly cooling magma body. Restart of magma influx restarts volcanic eruptions; pulsating magma supply (vs. continuous) has little overall effect on the state of the magma body at depth, which according to our modeling contains significant amount of melt.

## Methods

Zircons from three young (morphologically post-glacial, Holocene) lavas, one ~ 0.2 Ma Pleistocene lava, and 0.7 Ma ignimbrite were extracted and mounted in epoxy (see Fig. 2 for sample numbers). Samples were imaged by SEM using cathodoluminescence and backscattered electrons at the University of Alberta Canada and first studied by in-situ O isotope analysis with a CAMECA IMS 1280 ion microprobe using a Cs beam and ~ 15 µm diameter spots with a depth of ~ 1 µm. Mostly cores and some rims were targeted and the external precision was ± 0.2‰ (2σ) based on concurrently run standards. The same zircon crystals after a light repolishing were analyzed in overlapping spots for U–Th and U–Pb ages and then for Hf isotopes by LA–ICP–MS at the ETH Zurich using methods described in^47^ for U–Th and^48^ for U–Pb dating of young zircons. Samples were first analyzed by U–Th disequilibria and when ages were determined to be on equiline, zircons were additionally dated by U–Pb methods (Table S3 Supplementary). Lateral spot sizes were 30 µm with a crater depth of ~ 12 µm and 50 µm with a depth of ~ 18 µm for geochronological and Hf isotopic analysis, respectively. Errors for Hf isotopes were less than 1 epsilon unit.

In order to date the youngest zircon growth at high spatial resolution, selected zircon crystals from Elb5 and one additional sample Elb-18 were analyzed by secondary ion mass spectrometry (SIMS) U–Th depth profiling at the University of Heidelberg. Analyses of the outermost growth domains of zircon was carried out on unsectioned crystals pressed into indium metal so that prism faces were flush with the mount surface. SIMS analyses were carried out in multi-collection using the Heidelberg University CAMECA IMS 1280-HR following^44^ with the exception that four electron multipliers (EMs) were used and only masses between 244 and 254 were analyzed in 45 cycles total, each cycle consisting of three magnet jumps. U-Th relative sensitivity was calibrated using the method of^45^ by analyzing radiogenic ^208^Pb/^206^Pb on zircon references AS3 and 91500^49,50^. Accuracy of EM gain calibrations and background corrections for the unknowns was monitored by bracketing analyses of secular equilibrium zircon reference AS3 for which an average value of (^230^Th)/(^238^U) = 1.027 ± 0.010 (mean square of weighted deviates MSWD = 0.55; n = 10) was obtained during the analysis session. Due to the non-isochronous behavior of zircon in the investigated samples, two-point (zircon-melt) model isochrons were calculated using whole-rock Th and U abundances and assuming that the melt was in secular equilibrium. Several depth profiles indicated in-run age variability where model ages significantly increased with depth. To capture this age variability, all analyses were subdivided into three blocks, each block corresponding to an analysis depth of ~ 1 µm determined from measuring crater depth with a Bruker DektakXT stylus profilometer. For depth profiles where the MSWD calculated for three blocks exceeded the expected value of^51^, three separate ages are reported; for all other analyses, only one age averaging the entire ~ 3 µm deep analysis crater is computed (Fig. 3).

Whole rock samples were studied in thin section for phenocryst assemblage and proportion of crystals, by XRF and ICPMS for their major and trace elements and by laser fluorination at the University of Oregon, all analytical data is presented in the Supplementary Tables S1–S4.

### Numerical methods

Modeling methods included fine-tuning of the 2D dike and sill intrusion thermochemical model of Melnik et al.^41^, which utilizes the use of zircon growth and dissolution software published in^9^. The modeling effort computes temperature-melt percentage-time histories and growth and dissolution histories of individual model zircon crystals in ~ 10^5^ points across the entire magmatic system. Data is then extracted and post-processed. The goal of the modeling is to find the best conditions that match the Elbrus zircon record.

The employed program^41^ was adjusted to Elbrus initial and boundary conditions. The model is based on the solution of heat advection–diffusion equation accounting for latent heat release during magma crystallization, and implementing simplified T–X phase diagrams for magma and host rocks. Each parcel of magma enters the simulation domain as an elliptical body with a specified temperature and volume. Dikes are injected randomly within a specified area and are transferred from subvertical to subhorizontal (sills) bodies above a specified depth. Host rocks are assumed to be linearly elastic with a constant Young’s modulus and Poisson ratio. In this case, displacements of rocks and magma parcels are governed by an analytical solution^52^. In order to translate 2D magma flux to volumetric flux, we assume that the third dimension of the domain has a fixed length, and that the 2D approximation is valid for the central volcano in spite of absence of a preferred dike orientation common for extensive tectonic environments. The model allows for volcanic eruptions to occur as soon as the critical interconnected volume of eruptible magma accumulates beneath the volcano. We specify the exponential distribution of eruptive volumes following^42^ that leads to frequent small-size eruptions separated by long interruptive periods during which magma accumulates to be tapped in large eruptions. The model allows extraction of thermal histories of individual magma parcels that were erupted during particular episodes. Then, zircon growth of individual crystals is simulated by the model from^9^. The resulting zircon age distribution is calculated and compared with the measured ages. Parameters for the best fit scenario for Elbrus are listed in Table 1.

## Supplementary Information

Supplementary Figures.

Supplementary Tables.

## Data Availability

All data is available in Excel form as a supplementary to this paper.
