# Author Correction: Age of the magma chamber and its physicochemical state under Elbrus Greater Caucasus, Russia using zircon petrochronology and modeling insights

**DOI:** 10.1038/s41598-023-39242-y

**Published:** 2023-07-26

**Authors:** I. N. Bindeman, O. E. Melnik, M. Guillong, I. S. Utkin, J.-F. Wotzlaw, A. K. Schmitt, R. A. Stern

**Affiliations:** 1 Earth Sciences, University of Oregon, Eugene, OR USA; 2 Institute of Mechanics, Lomonosov Moscow State University, Moscow, Russia; 3 Institut des Sciences de la Terre (ISTerre), University Grenoble Alpes, Grenoble, France; 4 Department of Earth Sciences, ETH Zurich, Zurich, Switzerland; 5 Department of Civil, Environmental and Geomatic Engineering, ETH Zürich, Zurich, Switzerland; 6 Institut für Geowissenschaften, Universität Heidelberg, Heidelberg, Germany; 7 John de Laeter Centre, Curtin University, Bentley, WA Australia; 8 Canadian Centre for Isotopic Microanalysis, University of Alberta, Edmonton, Canada

Correction to: *Scientific Reports* 10.1038/s41598-023-36793-y, published online 15 June 2023

The original version of this Article contained errors.

The original version of this article contained an error in the name of I. S. Utkin which was incorrectly given as I. V. Utkin.

Furthermore, there was an error in Affiliation 5, which was incorrectly given as ‘Department of Earth Sciences, UNIL, Lausanne, Switzerland.’ The correct affiliation is listed below:

Department of Civil, Environmental and Geomatic Engineering, ETH Zürich, Zurich, Switzerland.

Finally, in the Acknowledgements section,

“The authors acknowledge the support of NSF grant EAR 1822977. OM was partly supported by the ERC Grant agreement 787399-SEISMAZE under the European Union Horizon 2020 Research and Innovation Programme.”

Now reads:

“The authors acknowledge the support of NSF Grant EAR 1822977. O.M. was partly supported by the ERC Grant agreement 787399-SEISMAZE and by the PAUSE program (Grant #C7H-PUB23A59.”

The original Article has been corrected.

