# Supplementary material for: Age of the magma chamber and its physicochemical state under Elbrus Greater Caucasus, Russia using zircon petrochronology and modeling insights
Source: Sci Rep. 2023 Jun 15;13:9733. doi: 10.1038/s41598-023-36793-y (PMC10272178; doi:10.1038/s41598-023-36793-y)
Supplement: Supplementary file 1 — Supplementary Figures. [file 41598_2023_36793_MOESM1_ESM.docx]

**Inventory of Supplementary Files**

*“Age of the magma chamber and its physicochemical state under Elbrus Greater Caucasus, Russia using zircon petrochronology and modeling insights” by*

*Bindeman IN, Melnik OE, Guillong M, Utkin IV, Wotzlaw J-F., Schmitt AK, Stern RA*

Supplementary Material consists of:

two Supplementary Figures Files and four Supplementary Tables S1-S4 (10 sheets) Supplementary Dataset with all data for studied samples

3

4

5

6

7

8

9

0

50

100

150

200

250

300

ln(n/V L), cm

-

-4

Zircon length,

µ

m

Fig. S1 Crystal size distribution of zircons in sample Elb5 showing lack of crystals less than 20 um and deficiency of small crystals, less than 60 μm. Vertical axis denotes abundance of zircons of certain size bin (L) per univ volume of magma, measured by weighing extracted amount of zircons from 50 g of rock using HF acid extraction


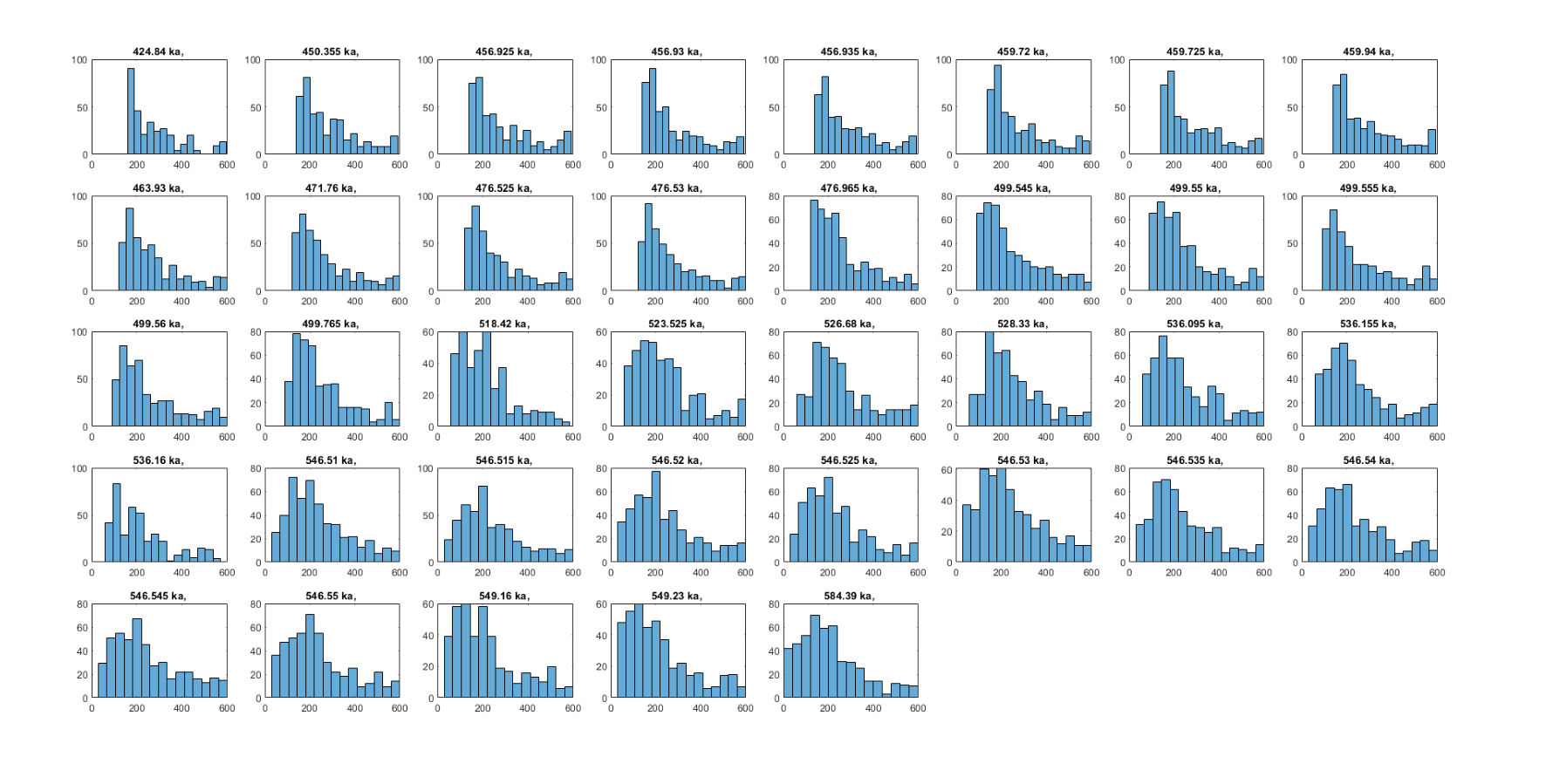

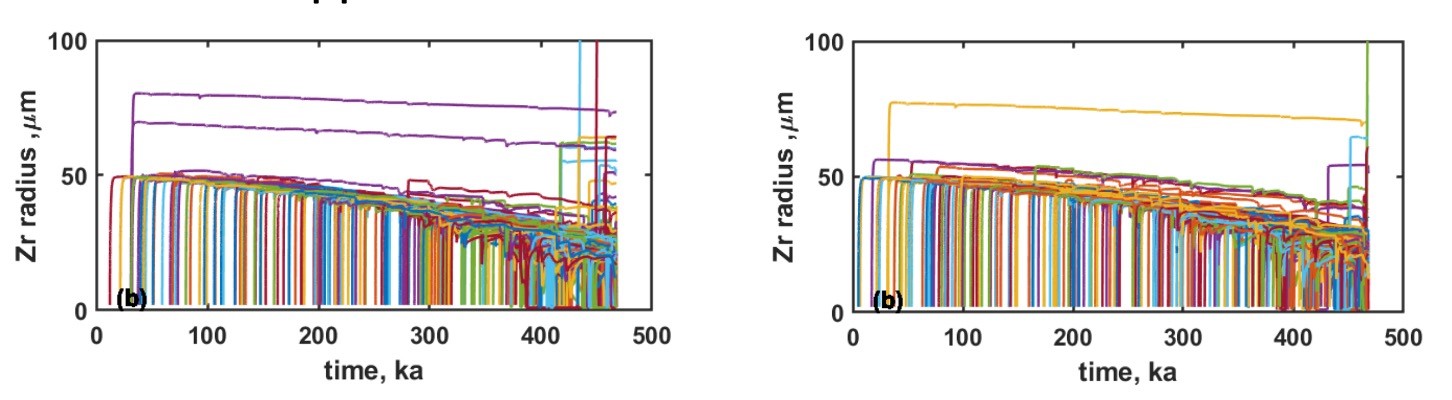


180

ppm Zr

190

ppm Zr

C

Fig. S2 A-B) destinies (growth-dissolution patterns) in zircons in different parts of the growing magmatic system with different Zr concentration in whole rock as sampled by the eruptions; notice that most zircons grow during cooling of dikes and then get dissolved, in line with age dating indicating that the minority of zircons of eruption age (Fig. 3), and with zircon CSDs (Fig. S1) indicating their preeruptive dissolution. C) Histograms of zircon age distributions in products of different eruptions, spanning the entire magma formation interval, compare to Fig. 2
